# Association of neighbourhood migrant density and risk of non-affective psychosis: a national, longitudinal cohort study

**DOI:** 10.1016/S2215-0366(20)30059-6

**Published:** 2020-04

**Authors:** Jennifer Dykxhoorn, Glyn Lewis, Anna-Clara Hollander, James B Kirkbride, Christina Dalman

**Affiliations:** a Division of Psychiatry, University College London, London, UK; b Department of Primary Care and Population Health, University College London, London, UK; c Department of Public Health Sciences, Karolinska Institutet, Stockholm, Sweden

## Abstract

**Background:**

Elevated risk of psychotic disorders in migrant groups is a public mental health priority. We investigated whether living in areas of high own-region migrant density was associated with reduced risk of psychotic disorders among migrants and their children, and whether generation status, probable visible minority status, or region-of-origin affected this relationship.

**Methods:**

We used the Swedish registers to identify migrants and their children born between Jan 1, 1982, and Dec 31, 1996, and living in Sweden on or after their 15th birthday. We tracked all included participants from age 15 years or date of migration until emigration, death, or study end (Dec 31, 2016). The outcome was an ICD-10 diagnosis of non-affective psychosis (F20–29). We calculated own-region and generation-specific own-region density within the 9208 small areas for market statistics neighbourhoods in Sweden, and estimated the relationship between density and diagnosis of non-affective psychotic disorders using multilevel Cox proportional hazards models, adjusting for individual confounders (generation status, age, sex, calendar year, lone dwelling, and time since migration [migrants only]), family confounders (family income, family unemployment, and social welfare), and neighbourhood confounders (deprivation index, population density, and proportion of lone dwellings), and using the Akaike information criterion (AIC) to compare model fit.

**Findings:**

Of 468 223 individuals included in the final cohort, 4582 (1·0%) had non-affective psychotic disorder. Lower own-region migrant density was associated with increased risk of psychotic disorders among migrants (hazard ratio [HR] 1·05, 95% CI 1·02–1·07 per 5% decrease) and children of migrants (1·03, 1·01–1·06), after adjustment. These effects were stronger for probable visible minority migrants (1·07, 1·04–1·11), including migrants from Asia (1·42, 1·15–1·76) and sub-Saharan Africa (1·28, 1·15–1·44), but not migrants from probable non-visible minority backgrounds (0·99, 0·94–1·04). Among migrants, adding generation status to the measure of own-region density provided a better fit to the data than overall own-region migrant density (AIC 36 103 *vs* 36 106, respectively), with a 5% decrease in generation-specific migrant density corresponding to a HR of 1·07 (1·04–1·11).

**Interpretation:**

Migrant density was associated with non-affective psychosis risk in migrants and their children. Stronger protective effects of migrant density were found for probable visible minority migrants and migrants from Asia and sub-Saharan Africa. For migrants, this risk intersected with generation status. Together, these results suggest that this health inequality is socially constructed.

**Funding:**

Wellcome Trust, Royal Society, Mental Health Research UK, University College London, National Institute for Health Research, Swedish Research Council, and FORTE.

## Introduction

Elevated psychosis risk in migrants and their children has been well established in European^2^, ^3^, ^4^, ^5^, ^6^ and North American studies,^7^, ^8^, ^9^ yet adequate explanations for this phenomenon have not been elucidated.^10^ The persistence of increased risk for children of migrants implicates factors in the post-migratory environment, including so-called ethnic density, in the development of psychotic disorders.^4^ The ethnic density hypothesis posits that minority ethnic individuals living in areas with higher proportions of people from their own ethnic group have better health outcomes than those living in areas with lower ethnic density.^11^ Some have theorised that this might be due to the protective effect of increased social support or fewer experiences of discrimination, although other explanations are possible.^12^, ^13^

Studies have observed an association between low ethnic density and elevated psychosis risk,^14^, ^15^, ^16^, ^17^, ^18^, ^19^, ^20^ yet the relationship between the two appears more nuanced than a simple association.^15^ For example, a study in the Netherlands found the difference between incidence rates in populations of low and high ethnic density was most pronounced for Moroccan migrants.^15^ A study in London (UK) found the highest schizophrenia risk among black and minority ethnic individuals who lived in areas with low ethnic density.^17^ These contextually specific effects might reflect the different migration patterns, attitudes to migrant reception, and meaning attached to ethnic identities in different contexts.^21^ Fewer studies have looked at migrant density. One study found a surprising association between migrant density and later risk of non-affective psychotic disorders among children of migrants but not among migrants.^1^ These findings indicate that the effect of migrant density on psychosis risk might vary by region of origin, ethnicity, probable visible minority status, or generation status; it is not yet clear what is underlying the observed patterns.

**Research in context**

**Evidence before this study**

Ethnic density—ie, the proportion of people from one's own ethnic group living in the immediate neighbourhood—has been proposed as a protective factor for psychotic disorders, but longitudinal evidence on this issue is sparse. We searched PubMed for studies published up to July 1, 2019, that measured the impact of ethnic density on psychotic disorders, using the terms “psychotic disorder*”, “schizophrenia”, “migrant density”, “ethnic density.” We identified 11 studies mostly published from cross-sectional studies in the UK or the Netherlands. While several studies suggested an overall association between greater ethnic density and reduced risk of psychotic disorders, this has not been shown for all ethnic groups. Only one set of studies from Denmark have considered whether ethnic density is longitudinally associated with non-affective psychotic disorders, finding an association for children of migrants but not migrants. Large, longitudinal, nationwide studies are required to determine whether these effects differ by generation status (ie, migrants versus their children), region of origin, visible minority status, or type of migrant density (ie, overall own-region migrant density or generation-specific own-region density).

**Added value of this study**

To our knowledge, this is the largest study to date investigating this issue, incorporating multilevel survival analysis to precisely model small area effects on psychosis risk. We show consistently increased risks of later psychotic disorders for migrants and children of migrants in neighbourhoods with lower own-region migrant density at age 15 years. This effect was particularly pronounced among probable visible minority migrant groups, including migrants from Asia and sub-Saharan Africa. We found no evidence for a migrant density effect for migrants or their children from other regions. Generation-specific migrant density was more strongly associated with psychosis risk in migrants, overall.

**Implications of all the available evidence**

Our longitudinal study strengthens earlier cross-sectional research by showing that own-group migrant density has stronger protective effects on non-affective psychosis risk for probable visible minority migrants. These effects were present for both migrants and their children, but were more pronounced by generation-specific density among migrants. Previous research has highlighted that ethnic density might be less important for some groups—eg, the black Caribbean population in the UK—than ethnic integration with respect to psychosis risk. This new evidence suggests that ethnic and migrant density might have intersectional effects with other factors involved in shaping psychosis risk. This research can be used to elucidate the pathways by which inequalities in mental health might be socially constructed, and creates an opportunity for public mental health intervention.

While it is likely that a broad range of factors underlie the migrant density effect, a plausible pathway by which migrant density affects psychotic disorder rates in migrants and their children could be through visible minority status. Those living in areas of low own-region migrant density might perceive themselves as different from others in their social environment, contributing to a sense of social exclusion, higher levels of social stress, and more frequent experiences of discrimination.^21^, ^22^ On the basis of these theories, we hypothesised that the migrant density effect would be more pronounced in probable visible minority than non-visible minority migrants.

While both migrants and their children might share visible minority status and could be subject to discriminatory experiences in the host country, it is probable that children of migrants have higher linguistic and cultural fluency in the host country. By contrast, first-generation migrants might rely on networks of individuals from the same region of origin who share language or cultural practices for social support, information, and connection to resources. Furthermore, neighbourhoods with high migrant density might be more likely to have culturally sensitive health and social services and access to religious facilities, ethnic foods, and cultural programmes. Thus, we hypothesised that the risk of psychotic disorders would be elevated for both migrants and their children living in areas of low migrant density, but that this effect would be more pronounced for migrants.

Here, we used prospectively collected registry data on a nationwide cohort in Sweden to examine how neighbourhood migrant density might influence subsequent risk of psychotic disorders, with consideration for the impact of generation status, probable visible minority status, and region of origin on the strength of this relationship.

## Methods

### Study design and population

This cohort study used data from Psychiatry Sweden, a comprehensive register linkage developed for mental health research^23^ that provides nationwide data on the entire population living in Sweden since 1920. It links together several registers, including those of most relevance here: the Register of the Total Population, the immigration and emigration register (known as STATIV), the Multi-Generation register, and the National Patient Register. The registers include all people living in Sweden, including immigrants from the time they are granted permanent residency rights. We identified all migrants (ie, individuals born outside of Sweden) and children of migrants (ie, individuals born in Sweden with at least one migrant parent) born between Jan 1, 1982, and Dec 31, 1996, and living in Sweden on or after their 15th birthday. Individuals were tracked from their 15th birthday or immigration to Sweden after age 15 years (earliest possible date: Jan 1, 1997) until emigration, death, or the end of the study period (Dec 31, 2016). We excluded temporary visitors and those without a residency permit, including asylum seekers and undocumented migrants; participants missing parental information, family income, and employment status; participants missing neighbourhood information at cohort entry or in the following year (to allow for register data to be updated by Statistics Sweden); and participants with an ICD-10 diagnosis of non-affective psychosis (F20–29) before their 15th birthday.

### Outcomes

Our outcome was an ICD-10 diagnosis of non-affective psychosis (F20–29) recorded in the National Patient Register. Date of cohort exit was defined as the date of first diagnosis from age 15 years or older, as this corresponds with the age of onset for psychotic disorders after which psychotic disorders can be reliably captured by diagnostic criteria in the Swedish health-care system.^24^

### Exposures

We estimated own-region migrant density for migrants and their children by using the 9208 small areas for market statistics (SAMS) neighbourhoods maintained by Statistics Sweden; the median population size of a SAMS neighbourhood in 2011 was 726 people (IQR 312–1378). We determined the total population in each neighbourhood by migrant status and region of origin, and estimated SAMS area-level characteristics including our migrant density exposures. We considered the SAMS neighbourhoods in which migrants lived at age 15 years or after immigration to Sweden, if later. Full details on how we derived our migrant density variables are given in the appendix (p 2).

We considered eight regions of origin: Nordic, Europe (excluding Nordic countries), Asia, Oceania, Middle East and north Africa, sub-Saharan Africa, North America (including Mexico), and South America. We included two additional categories—mixed migrant or Swedish migrant—for children of migrants where patients were from different regions: children of migrants were classified as Swedish migrant if they had one Swedish parent and one migrant parent, or were classified as mixed migrant if they had two migrant parents from different regions.

We estimated two migrant density exposures: overall own-region migrant density and generation-specific own-region migrant density. Overall own-region migrant density was estimated as the percentage of the neighbourhood total population from the same region of origin as the migrants in question, including both migrants and children of migrants. For example, for a migrant (or child of migrant) from Asia, this would be the proportion of the neighbourhood population who were either migrants or children of migrants from Asia. Generation-specific migrant density was restricted to the proportion of people from each participant's own region and generation status (ie, migrant or children of migrant). For example, for a migrant from Asia, this would be the proportion of the neighbourhood population who were also migrants from Asia; for children of migrants from Asia, this would be the proportion of the neighbourhood population who were also children of migrants from Asia. For both exposures, we calculated quintiles of migrant density and a continuous measure (5% change).

Probable visible minority density combined those from Asia, the Middle East and north Africa, sub-Saharan Africa, and South America, and probable non-visible minorities were individuals from Nordic countries, Europe, Oceania, and North America. Our classification of participants according to this definition of probable visible minority status was based on our understanding of the majority ethnicities in each participant's region of origin. We expressed this as 5% change in the proportion of the neighbourhood population who were probable visible minorities.

### Covariates

We included several individual and family covariates: sex, age, calendar year, generation status, lone dwelling, time since migration (migrants only), family disposable income quintile, receipt of social welfare, and family unemployment (of all family members in the same household). Age was modelled as a time-varying covariate because risk of psychotic disorders varies substantially by age,^25^ and all other covariates were included as fixed covariates. Any household with only one individual registered at cohort entry was considered a lone dwelling household. Disposable family income quintiles were calculated on the basis of the total population in each year, and families were categorised from quintile 1 (lowest) to quintile 5 (highest). Each individual was assigned their familial income quintile in the year of their cohort entry. Familial receipt of social welfare and unemployment status were defined as binary variables (receiving social welfare benefits *vs* not receiving and any family member unemployed *vs* employed, respectively).

For each neighbourhood, for each year, we calculated population density (people per km^2^), proportion of lone dwelling households, and a deprivation index score. The deprivation score was generated by calculating the proportion of each neighbourhood which was low income, unemployed, receiving social welfare, or convicted of a criminal offence. These proportions were standardised and summed to create quintiles of deprivation from quintile 1 (least deprived) to quintile 5 (most deprived). Participants were assigned values of these neighbourhood covariates in their year of cohort entry, as for the migrant density variables above.

### Statistical analysis

We inspected the level of missingness in the data, which was low (6·0%) overall and therefore conducted a complete-case analysis, as it was expected to produce unbiased results.^29^ We used multilevel Cox proportional hazards regression, accounting for the hierarchical structure of the data (participants were nested within neighbourhoods). We used Mestreg in Stata to fit a random-effects Weibull model with normally distributed random effects, which allowed the baseline hazard to vary across neighbourhoods.

The null model, without fixed effects, was fitted to quantify the variation in the baseline hazard for psychosis attributable to the neighbourhood level, assessed via Wald χ^2^ tests. Next, we fitted an unadjusted model including each migrant density exposure separately as a predictor of psychosis incidence. We then adjusted for individual confounders (generation status, age, sex, calendar year, lone dwelling, and time since migration [migrants only]), family confounders (family income, family unemployment, and social welfare), and neighbourhood confounders (deprivation index, population density, and proportion of lone dwellings) in separate models, before fitting a fully adjusted model. To investigate whether overall own-region migrant density or generation-specific migrant density fitted the data better for migrants and children of migrants, we estimated stratified fully adjusted models for migrants and children of migrants separately. The Akaike information criterion (AIC) was calculated to compare model fit, where better fit was indicated by lower scores. We then investigated whether own-region migrant density had a different effect on psychosis risk by probable visible minority status. Finally, we accounted for region of origin to ensure our density measures were not merely a proxy for region. We adjusted our models for region and calculated region-specific migrant density effects in a supplemental analysis.

We present descriptive statistics of the cohort, including percentages and median (IQR), and the Pearson correlation coefficient to compare correlation between migrant density measures. We also report unadjusted and adjusted hazard ratios (HRs) with 95% CIs. We used Stata (version 15.1) for all analyses.

### Role of the funding source

The funders of the study had no role in study design, data collection, data analysis, data interpretation, or writing of the report. The corresponding author had full access to all the data in the study and had final responsibility for the decision to submit for publication.

## Results

498 340 participants were eligible for inclusion, of whom 30 117 had missing data. Most missing data regarded family income, with 4·5% of participants missing family income information (figure; appendix p 3).

**Figure:**
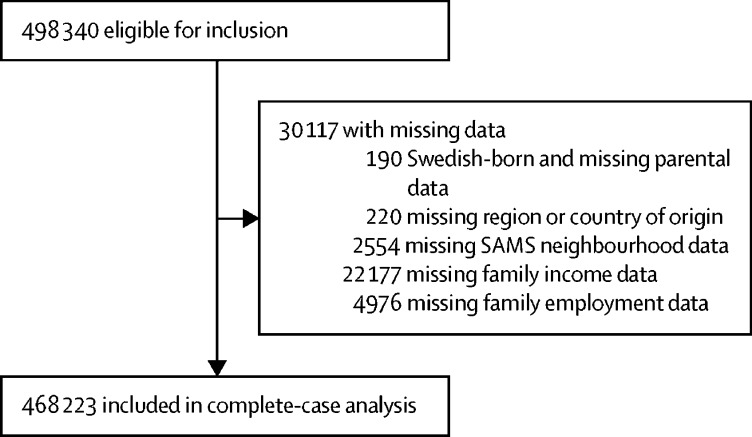
Study profile

468 223 individuals were included in the final cohort for our complete-case analysis (figure): 268 686 (57·4%) were migrants and 199 537 (42·6%) were children of migrants. The cohort included 4582 cases (1·0%) of non-affective psychotic disorder with 5·0 million person-years of follow-up. The largest group of migrants were from Europe, excluding Nordic countries, whereas the largest proportion of children of migrants were of Nordic origin (table 1).

**Table 1:** Individual-level and neighbourhood-level sociodemographic characteristics

	**Migrants (n=268 686)**			**Children of migrants (n=199 537)**		
	Percentage of migrants	Cases*	Person-years in dataset	Percentage of children of migrants	Cases*	Person-years in dataset
**Non-affective psychosis**						
Yes	0·9%	2468	17 902	1·1%	2114	17 374
No	99·1%	266 218	2 585 051	98·9%	197 423	2 398 031
**Sex**						
Female	49·2%	845 (34·2%)	1 281 490	48·6%	752 (35·6%)	1 170 667
Male	50·8%	1623 (65·8%)	1 321 463	51·4%	1362 (64·4%)	1 244 738
**Date of birth**						
1982–86	52·6%	1358 (55%)	1 450 168	25·8%	776 (36·7%)	881 907
1987–92	31·2%	790 (32%)	823 312	34·0%	801 (37·9%)	861 980
1993–96	16·3%	320 (13%)	329 473	40·2%	537 (25·4%)	671 518
**Region**†						
Nordic‡	7·7%	103 (4·2%)	131 882	28·7%	644 (30·5%)	766 149
Europe	31·8%	693 (28·1%)	880 211	13·5%	270 (12·8%)	310 934
Asia	16·4%	297 (12%)	365 971	4·1%	61 (2·9%)	92 699
Oceania	0·4%	3 (0·1%)	7648	0·0%	0	62
Middle East and north Africa	27·3%	693 (28·1%)	796 928	20·9%	349 (16·5%)	471 962
Sub-Saharan Africa	10·5%	550 (22·3%)	261 899	3·8%	127 (6%)	72 516
North America	2·5%	50 (2%)	55 558	0·3%	6 (0·3%)	6338
South America	3·4%	79 (3·2%)	102 857	3·5%	66 (3·1%)	84 025
Swedish migrant	..	..	..	18·9%	390 (18·4%)	456 995
Mixed migrant	..	..	..	6·4%	201 (9·5%)	153 726
**Family income**						
Quintile 1 (lowest)	49·1%	848 (34·4%)	958 143	4·1%	99 (4·7%)	80 303
Quintile 2	15·1%	422 (17·1%)	432 327	13·6%	388 (18·4%)	315 374
Quintile 3	19·2%	650 (26·3%)	640 135	25·6%	671 (31·7%)	685 210
Quintile 4	11·3%	386 (15·6%)	398 985	29·8%	582 (27·5%)	727 617
Quintile 5 (highest)	5·3%	162 (6·6%)	173 363	24·0%	374 (17·7%)	606 902
**Family receiving social welfare**						
Yes	34·4%	1299 (52·6%)	1 090 802	16·9%	573 (27·1%)	405 858
No	65·6%	1169 (47·4%)	1 512 152	83·1%	1541 (72·9%)	2 009 547
**Family unemployment**						
Yes	10·4%	365 (14·8%)	393 276	24·8%	608 (28·8%)	624 504
No	89·6%	2103 (85·2%)	2 209 677	75·2%	1506 (71·2%)	1 790 901
**Own-region migrant density**						
Quintile 1 (lowest)	19·2%	604 (24·5%)	510 823	15·5%	391 (18·5%)	374 540
Quintile 2	18·8%	474 (19·2%)	494 666	22·4%	462 (21·9%)	544 363
Quintile 3	19·4%	409 (16·6%)	472 935	22·2%	447 (21·1%)	531 915
Quintile 4	20·7%	464 (18·8%)	522 679	20·8%	420 (19·9%)	515 082
Quintile 5 (highest)	21·9%	517 (20·9%)	601 851	19·1%	394 (18·6%)	449 505
**Generation-specific migrant density**§						
Quintile 1 (lowest)	16·2%	524 (21·2%)	431 697	20·0%	477 (22·6%)	469 565
Quintile 2	20·3%	498 (20·2%)	507 747	20·0%	399 (18·9%)	480 988
Quintile 3	20·9%	478 (19·4%)	533 552	20·0%	404 (19·1%)	491 161
Quintile 4	21·3%	460 (18·6%)	550 091	20·0%	415 (19·6%)	491 463
Quintile 5 (highest)	21·3%	508 (20·6%)	579 867	20·0%	419 (19·8%)	482 228

* Percentages are given on number of cases.

† Region of birth for migrants and region of parental birth for children of migrants.

‡ Includes children of migrants with one Nordic-born parent and one Swedish-born parent.

§ Migrants from same region or children of migrants from same parental region.

Levels of neighbourhood migrant density varied by neighbourhood, participant region of origin, and generation status. Overall, median own-region migrant density was 6·4% (IQR 3·0–13·0; range 0·0–80·0; appendix p 4). The range of possible values of own-region density varied by region of origin. For migrants and children of migrants from Oceania, the maximum neighbourhood own-region density was 1·8%, whereas those from the Middle East and north Africa could live in a neighbourhood with up to 80·0% of the neighbourhood from the same region of origin.

Separated by generation status, median neighbourhood own-region migrant density was 4·9% (IQR 2·2–10·6; range 0·0–58·7) for migrants and 3·4% (1·5–5·6; 0·0–35·5) for children of migrants (appendix p 4).

A null multilevel Cox regression model showed that some of the variance in psychosis rates was explained by neighbourhood-level clustering (Wald χ^2^ p=0·0001; appendix p 4), justifying use of a multilevel approach for this analysis. The unadjusted estimates showed that each 5% decrease in own-region migrant density was accompanied by a 3% increase in risk of developing psychotic disorders (HR 1·03, 95% CI 1·02–1·05; table 2). After adjustment for individual, family, and neighbourhood confounders, the effect remained, such that a 5% decrease in own-region migrant density corresponded to a 5% increase in psychosis risk (1·05, 1·03–1·06; table 2). When investigated by quintiles of migrant density, both unadjusted and adjusted estimates showed an increase in risk of psychotic disorders as own-region migrant density decreased (table 2).

**Table 2:** Hazard ratios of non-affective psychosis by quintiles of own-region migrant density

		**Hazard ratio (95% CI)**	**AIC**
Unadjusted		..	72 817
	Quintile 1 (lowest)	1·35 (1·23–1·48)*	..
	Quintile 2	1·07 (0·97–1·18)	..
	Quintile 3	1·01 (0·92–1·12)	..
	Quintile 4	1·00 (0·91–1·11)	..
	Quintile 5 (highest)	1 (ref)	..
Individual-adjusted and family-adjusted		..	72 767
	Quintile 1 (lowest)	1·35 (1·23–1·48)*	..
	Quintile 2	1·13 (1·03–1·24)*	..
	Quintile 3	1·10 (0·99–1·21)	..
	Quintile 4	1·05 (0·96–1·16)	..
	Quintile 5 (highest)	1 (ref)	..
Neighbourhood-adjusted only		..	72 715
	Quintile 1 (lowest)	1·60 (1·44–1·77)*	..
	Quintile 2	1·26 (1·13–1·40)*	..
	Quintile 3	1·17 (1·05–1·30)*	..
	Quintile 4	1·12 (1·01–1·24)*	..
	Quintile 5 (highest)	1 (ref)	..
Fully adjusted		..	69 952
	Quintile 1 (lowest)	1·36 (1·22–1·52)*	..
	Quintile 2	1·14 (1·03–1·27)*	..
	Quintile 3	1·11 (1·00–1·23)	..
	Quintile 4	1·07 (0·96–1·18)	..
	Quintile 5 (highest)	1 (ref)	..
Per 5% decrease in density			
	Unadjusted	1·03 (1·02–1·05)*	72 799
	Individual-adjusted only	1·02 (1·01–1·04)*	69 964
	Neighbourhood-adjusted only	1·07 (1·05–1·09)*	72 741
	Fully adjusted^†^	1·05 (1·03–1·06)*	69 957

Models were adjusted for individual and family confounders (generation status, age, sex, calendar year, lone dwelling, family income, social welfare, family unemployment, and time since migration [migrants only]) or neighbourhood confounders (deprivation index, population density, and proportion of lone dwellings). Fully adjusted estimates include individual, family, and neighbourhood confounders. AIC=Akaike information criterion.

* p<0·05.

The correlation between own-region migrant density and generation-specific migrant density was high (correlation 0·90), and there was a similar pattern of risk for migrants regardless of the measure used (table 3). A 5% decrease in own-region density corresponded to a 5% elevation in risk of non-affective psychosis in migrants (HR 1·05, 95% CI 1·02–1·07) whereas a 5% decrease in generation-specific density corresponded to a 7% elevation (1·07, 1·04–1·11; table 3). Among children of migrants, a 5% decrease in own-region density corresponded to a 3% increase in risk (1·03, 1·01–1·06). When comparing the model fit for these two measures of migrant density, we found that the generation-specific measure described the data better for migrants, with a lower AIC, but that the own-region measure performed better for children of migrants (table 3).

**Table 3:** Fully adjusted hazard ratios of non-affective psychotic disorders and migrant density, by generation status

		**Migrants**		**Children of migrants**	
		Adjusted hazard ratio (95% CI)	AIC	Adjusted hazard ratio (95% CI)	AIC
Own-region migrant density		..	36 110	..	33 307
	Quintile 1 (lowest)	1·37 (1·17–1·59)*	..	1·28 (1·10–1·49)*	..
	Quintile 2	1·17 (1·00–1·36)	..	1·10 (0·95–1·28)	..
	Quintile 3	1·12 (0·97–1·30)	..	1·08 (0·93–1·25)	..
	Quintile 4	1·13 (0·99–1·30)	..	1·01 (0·87–1·17)	..
	Quintile 5 (highest)	1 (ref)	..	1 (ref)	..
	Per 5% decrease	1·05 (1·02–1·07)*	36 106	1·03 (1·01–1·06)*	33 307
Generation-specific own-region migrant density		..	36 102	..	33 311
	Quintile 1 (lowest)	1·42 (1·21–1·67)*	..	1·16 (1·01–1·34)*	..
	Quintile 2	1·17 (1·01–1·37)*	..	0·96 (0·84–1·11)	..
	Quintile 3	1·12 (0·96–1·29)	..	1·00 (0·87–1·15)	..
	Quintile 4	1·04 (0·90–1·20)	..	1·02 (0·89–1·18)	..
	Quintile 5 (highest)	1 (ref)	..	1 (ref)	..
	Per 5% decrease	1·07 (1·04–1·11)*	36 103	1·03 (0·97–1·09)	33 313
Own-region migrant density by visible minority status (per 5% decrease)					
	Probable visible minorities	1·07 (1·04–1·11)*	..	1·04 (1·00–1·08)	..
	Probable non-visible minorities	0·99 (0·94–1·04)	..	0·99 (0·96–1·03)	..

Hazard ratios are adjusted for individual and family confounders (age, sex, lone dwelling, family income, social welfare, family unemployment, and time since migration [migrants only]) and neighbourhood confounders (deprivation index, population density, and proportion of lone dwellings). AIC=Akaike information criterion.

* p<0·05.

Among probable visible minority individuals, the risk of psychosis increased by 7% per 5% decrease in own-region migrant density (HR 1·07, 95% CI 1·04–1·09) for migrants and children of migrants combined. There was no evidence of an increase in risk of psychosis diagnosis among non-visible minority migrants (1·00, 0·97–1·02). The increased risk for probable visible minority individuals was slightly stronger among migrants than children of migrants (table 3).

When region was added to the fully adjusted analysis, the point estimates for migrant density effect followed a similar pattern, but precision was lower and 95% CIs overlapped unity (appendix p 5). In the region-adjusted model, there was evidence of a null effect among children of migrants (HR 1·00, 95% CI 0·97–1·03).

We observed differences in the migrant density effect by region of origin. We found increased risk of non-affective psychosis for migrants from Asia and sub-Saharan Africa in areas of lower migrant density (table 4), but no evidence of a migrant density effect for other migrant groups or for children of migrants from any specific region; however, we cannot exclude uncertainty due to low power in some subgroups.

**Table 4:** Region-specific migrant density effects, by generation status

	**Migrants**			**Children of migrants**		
	Cases	Person-years	Adjusted hazard ratio (95% CI)	Cases	Person-years	Adjusted hazard ratio (95% CI)
Nordic*	103	131 882	1·01 (0·90–1·13)	644	766 149	0·97 (0·93–1·00)
Europe	693	880 211	0·98 (0·92–1·04)	270	310 934	1·06 (0·96–1·17)
Asia	297	365 971	1·42 (1·15–1·76)†	61	92 699	1·15 (0·73–1·79)
Middle East and north Africa	693	796 928	1·03 (0·98–1·08)	349	471 962	1·00 (0·94–1·06)
Sub-Saharan Africa	550	261 899	1·28 (1·15–1·44)†	127	72 516	0·94 (0·80–1·11)
North America	50	55 558	1·77 (0·21–14·77)	6	6338	0·89 (0·00–162·16)
South America	79	102 857	0·67 (0·43–1·05)	66	84 025	1·15 (0·69–1·93)
Swedish migrant	..	..	..	390	456 995	0·83 (0·59–1·18)
Mixed migrant	..	..	..	201	153 726	1·17 (0·33–4·10)

Numbers were too low in the Oceania group for the model to converge, so excluded from this analysis. Hazard ratios were adjusted for individual and family confounders (age, sex, lone dwelling, family income, social welfare, family unemployment, and time since migration [migrants only]) and neighbourhood confounders (deprivation index, population density, and proportion of lone dwellings) and are calculated per 5% decrease in density.

* Includes children of migrants with one Nordic-born parent and one Swedish-born parent.

† p<0·05.

## Discussion

In this national, longitudinal cohort study, we showed that risk of non-affective psychotic disorder among migrants in Sweden increased as neighbourhood own-region migrant density decreased. We found this effect for both migrants and children of migrants. The impact of own-region migrant density was more pronounced for probable visible minority individuals than probable non-visible minority migrants. Additionally, we showed that consideration of generational migrant density status (ie, migrants or children of migrants) was important for migrants, but overall own-region migrant density described the data better for children of migrants.

Our findings should be considered in the context of several limitations. First, we had no information on ethnicity, which is not routinely collected in Swedish registers. We used region of origin to estimate migrant density, which might not align with an individual's self-reported ethnicity.^26^, ^27^, ^28^ Furthermore, our definition of probable visible minority status was based on our knowledge of the majority ethnicity in each region of origin, which might have led to measurement error without direct information on participants' ethnicity. In all likelihood, a small proportion of individuals were misclassified according to probable visible minority status. It is possible that factors other than visible minority status align with our classification of migrants by this measure, including level of economic development in their region of origin, cultural factors, or other reasons hitherto unknown.

Second, there were some missing data on exposures and covariates. We did a complete-case analysis, as it was expected to produce unbiased results with these modest levels of missing data.^29^

Third, differential use of the health-care system by migrant density might have biased the results of this study. Previous research has shown that migrants use psychiatric care at lower rates than Swedish-born individuals.^30^ It is plausible that in neighbourhoods with high migrant density, psychiatric services are underused and thus diagnoses are less common. Underuse in areas of high migrant density could be due to lower health literacy, lack of culturally sensitive services, reduced familiarity with the health-care system, or higher levels of mental health stigma and corresponding hesitation to access care.^31^, ^32^ Neighbourhood stratification of public infrastructure could result in areas with fewer health-care options, reduced access to specialised psychiatric services, and lower quality of care in more deprived neighbourhoods,^33^ which might differentially affect migrants or minority ethnic populations.

Finally, there were limitations of the spatially referenced data used in this study, including issues of scale and multiple addresses. Previous research has used a range of geographical regions to determine spatial boundaries, but there is little evidence showing at which scale geographical factors have salience.^34^ Furthermore, although the registered address could be where a migrant resides, they might spend substantial portions of time in different neighbourhood environments; as we do not have a measure of time spent in other spaces, we also do not know their exposure to migrant density in those spaces.^34^ Neighbourhood factors were measured at a single point in time and thus do not capture the cumulative exposures over time or differing exposure levels for those who have moved.^35^ Our multilevel analysis accounting for neighbourhood clustering represents an advance over previous research; however, further research using spatial modelling techniques would enhance our understanding of how individuals experience spatial exposures.

This study has several strengths, including nearly complete coverage in Sweden for 19 years of follow-up, including migrants arriving between 1982 and 2011. This ensured we included several important waves of immigration to Sweden of both labour migrants and refugees from diverse settings, such as Iraq, Iran, Afghanistan, and eastern Africa.^36^ Our long follow-up period, until December, 2016, allowed us to investigate migrant density throughout Sweden. The choice of a multilevel approach correctly modelled dependencies within the data in a survival context for the first time, to our knowledge, which is a notable advance on previous research. A wide range of confounders were considered and both own-region and generation-specific migrant density were investigated, which were measured prospectively in relation to the outcome.

Previous research has shown an overall migrant density effect for psychotic disorders^14^, ^15^, ^16^, ^17^, ^18^, ^19^, ^20^ and psychotic experiences,^37^ but that the effect was concentrated in certain migrant groups, including black African and black Caribbean groups.^37^, ^38^, ^39^ Consistent with these studies, we found an overall migrant density effect, with stronger evidence for migrants from Asia and sub-Saharan Africa than other groups.

Our finding that the migrant density effect was similar in migrants and children of migrants contradicts a Danish study that found stronger neighbourhood ethnic density effects for children of migrants than migrants.^1^ There were methodological differences between the studies that might explain these disparate results. The Danish study was based on a smaller cohort of 90 476 migrants and their children, compared with the 468 223 included in our study. The Danish study used parish areas (3500 residents) whereas our study used smaller neighbourhoods (median of 726 residents). There were some differences in how we classified regions and calculated migrant density. The Danish study did not account for the multilevel nature of these data in the analysis. These divergent results could be partially explained by different national approaches to migration and integration in Sweden and Denmark; however, these countries are similar in other ways, so this is unlikely to fully account for the observed differences. Considering these contrasting results by generation status, more research is needed to reach a consensus.

There are many aspects of the neighbourhood that could explain elevated risk of psychotic disorders in some migrant groups. Previous research has highlighted the importance of population density and neighbourhood deprivation as explanatory factors for elevated psychotic disorders in migrant groups. As migrants are more likely to reside in urban areas, and urbanicity has been linked to increased rates of psychotic disorders,^40^ we controlled for population density. Neighbourhood deprivation could also play a role in the patterning of psychosis risk, as residential segregation by socioeconomic status and ethnicity is persistent in many cities,^13^ and migrants and minority ethnic groups are more likely to live in deprived neighbourhoods owing to limited socioeconomic resources and structural racism.^41^, ^42^, ^43^ Thus, we controlled for neighbourhood deprivation to ensure the propensity for migrant groups to reside in more deprived neighbourhoods did not account for our findings.

It is plausible that the observed migrant density effect is due to aspects of the neighbourhood social environment, including the impact of social support, social stress, experiences of inclusion or exclusion, and experiences of discrimination and racism. Migrant density might work through psychosocial pathways^12^ to provide increased social support, enhance feelings of inclusion, and buffer individuals from experiences of racism and discrimination. While family members comprise an important source of social support and social capital,^44^ relationships with friends and neighbours can contribute to a wider sense of inclusion and belonging. Migrants moving to areas with a high concentration of individuals from the same region could experience a greater sense of belonging, feel connected to others, and have access to emotional and practical support.^45^ Furthermore, living in areas of low ethnicity density could lead to the perception of being different from one's social environment, contributing to higher levels of social stress, and more frequent experiences of discrimination.^21^, ^22^ Previous research has shown lower rates of reported discrimination and low social support among minority ethnic individuals living in areas of high migrant density.^38^ The direct experience of discrimination has been shown to affect health, but also the fear of racisim^46^ and perceived discrimination^47^ can have detrimental effects on mental health. Our findings of a protective migrant density effect among probable visible minority migrants could be due to reduced exposure to racism, discrimination, or feelings of isolation and otherness, although this was not found for children of migrants.^22^, ^48^

Our results align with a proposed neurobiological pathway to psychotic disorders via alterations to threat perception.^47^, ^49^, ^50^, ^51^ Using functional MRI, McCutcheon and colleagues^52^ showed increased amygdala responses to outgroup faces for both black and white ethnic groups, with evidence that this was more pronounced for those from residential areas with low own-region ethnicity. Minority ethnic groups, particularly those living in areas of low own-region migrant density, might have more frequent exposure to outgroup faces, corresponding to heightened amygdala responses.^53^ The greater outgroup contact in day-to-day life for visible minority individuals and the neurobiological response to threat experiences are consistent with our observation of highest risk among probable visible minority living in areas of low migrant density.

In conclusion, we found evidence that migrant density reduced the risk of psychotic disorders for migrants and children of migrants. Generation-specific density was more strongly associated with reduced risk of psychotic disorder for migrants, for whom having neighbours with a shared migration experience, language, or culture could be important. For children of migrants who were born in Sweden and thus face fewer linguistic and cultural barriers to integration than their parents, generation-specific density was less important than overall migrant density. The protective effect of migrant density was pronounced for probable visible minority migrants and children of migrants but not for probable non-visible minority individuals. While these findings largely align with previous studies, this study shows unique patterns of risk by generation status; further research is warranted to understand the underlying mechanisms of the migrant density effect.
