# Supplementary material for: Association of neighbourhood migrant density and risk of non-affective psychosis: a national, longitudinal cohort study
Source: Lancet Psychiatry. 2020 Apr;7(4):327–36. doi: 10.1016/S2215-0366(20)30059-6 (PMC7083220; doi:10.1016/S2215-0366(20)30059-6)
Supplement: Supplementary appendix [file mmc1.pdf]

THE LANCET
Psychiatry

Supplementary appendix

This appendix formed part of the original submission and has been peer reviewed. We post it as supplied by the authors.

Supplement to: Dykxhoorn J, Lewis G, Hollander A-C, Kirkbride JB, Dalman C. Association of neighbourhood migrant density and risk of non-affective psychosis: a national, longitudinal cohort study. *Lancet Psychiatry* 2020; published online March 5. https://doi.org/10.1016/S2215-0366(20)30059-6.

Table of Contents

Details for calculation of migrant density variables 2

Table 1 Total sample and complete case sample, overall and by generation 3

Table 2 Distribution of the migrant density proportion across neighbourhoods 4

Table 3 Percent (%) migrants & children of migrants in each quintile of own-group migrant density, by region 4

Table 4 Neighbourhood level random effects 4

Table 5 Total sample and complete case sample, overall and by generation 5

Table 6 Strengthening the Reporting of Observational Studies in Epidemiology (STROBE) checklist for cohort studies 5

Details for calculation of migrant density variables

We utilized the 9,208 “Small Areas for Market Statistics” (SAMS) neighbourhoods maintained by Statistics Sweden. Each individual living in Sweden must register their address when they immigrate to Sweden or subsequently move house.[1] This allowed us to determine the total population in each neigbhourhood by migrant status and region of origin, and estimate SAMS area-level characteristics including our migrant density exposures.

To permit valid estimates of migrant density, any neighbourhood with fewer than 50 people registered in 2011 was combined with its nearest neighbors using ArcGIS 10·5 until the minimum total population size was achieved, resulting in 8,047 neighbourhoods. For each neighbourhood, for each year (1997-2011), we obtained the total population size stratified by region-of-origin from the Register of the Total Population, divided into 8 categories: Nordic, Europe (excluding Nordic countries), Asia, Oceania, Middle East + North Africa, Sub-Saharan Africa, North America, and South America. Two additional categories were included for children of migrants where parents were from two different regions: (1) one Swedish parent and one migrant parent were classified as “Swedish-migrant”; (2) Two migrant parents from different regions were considered “mixed-migrant”. Probable visible minority density combined those from Asia, the Middle East, North Africa, Sub-Saharan Africa, and South America, and probable non-visible minorities were individuals from Nordic countries, Europe, Oceania, and North America. We selected these regions based on the assumption that migrants and children of migrants from these regions were probable visible minorities within Sweden. We excluded those with mixed ancestry from the analysis by probable visible minority status, as there would likely be higher misclassification within this group.

References

1. Sundberg P. *The Swedish address system*. Lantmäteriverket, Stockholm, Sweden; 2010.

Table 1 Total sample and complete case sample, overall and by generation

OVERALL		Total n	(n= 498,340) %	Complete case (n=468,223) n	%	Chi² (df); p-value [c]
Sex						
	Female	244,070	49.0	229,045	48.9	0.33 (1);
	Male	254,270	51.0	239,178	51.1	p=0.56
Date of birth						
	1982-1986	202,219	40.6	192,680	41.2	68 (2);
	1987-1992	158,265	31.8	151,508	32.4	p<0.001
	1993-1996	134,892	27.1	124,035	26.5	
	Missing	2,964	0.6	0	0.0	
Region[a]						
	Nordic[b]	79,600	16.0	78,012	16.7	1,100(9);
	Europe	117,839	23.7	112,384	24.0	p<0.001
	Asia	62,970	12.6	52,234	11.2	
	Oceania	1,196	0.2	1,132	0.2	
	Middle East + North Africa	118,182	23.7	115,047	24.6	
	sub-Saharan Africa	38,143	7.6	35,699	7.6	
	North America	7,971	1.6	7,279	1.6	
	South America	21,096	4.2	15,985	3.4	
	Unknown	220	0.0	0	0	
	Swedish-migrant	37,917	7.6	37,605	8.0	
	Mixed migrant	12,952	2.6	12,846	2.7	
	Swedish-born, no parents	254	0.0	0	0.0	
**MIGRANTS**		**Total**	**(n=296,895)**	**Complete case (n=268,686)**		
Sex						
	Female	146,140	49.2	132,057	49.2	0.31 (1);
	Male	150,755	50.8	136,629	50.8	p=0.58
Date of birth						
	1982-1986	150,624	50.7	141,204	52.6	427 (2)
	1987-1992	90,315	30.4	83,695	31.2	p<0.001
	1993-1996	54,239	18.3	43,787	16.3	
	Missing	1,717	0.6	0	0.0	
Region[a]						
	Nordic[b]	22,080	7.4	20,785	7.7	1,200 (7)
	Europe	90,677	30.5	85,553	31.8	p<0.001
	Asia	54,737	18.4	44,050	16.4	
	Oceania	1,190	0.4	1,126	0.4	
	Middle East + North Africa	76,115	25.6	73,298	27.3	
	sub-Saharan Africa	30,435	10.3	28,139	10.5	
	North America	7,394	2.5	6,708	2.5	
	South America	14,047	4.7	9,027	3.4	
	Unknown	220	0.1	0	0.0	
**Children of migrants**		**Total**	**(n 201,191)**	**Complete case (n=199,537)**		
Sex						
	Female	97,804	48.6	96,988	48.6	0.001 (1);
	Male	103,387	51.4	102,549	51.4	p=0.97
Date of birth						
	1982-1986	51,595	25.6	51,476	25.8	0.20 (2);
	1987-1992	67,950	33.8	67,813	34.0	p=0.90
	1993-1996	80,653	40.1	80,248	40.2	
	Missing	993	0.5	0	0.0	
Region[a]						
	Nordic[b]	57,520	28.6	57,227	28.7	1.1 (9);
	Europe	27,162	13.5	26,831	13.4	p=1.00
	Asia	8,233	4.1	8,184	4.1	
	Oceania	6	0.0	6	0.0	
	Middle East + North Africa	42,067	20.9	41,749	20.9	
	sub-Saharan Africa	7,708	3.8	7,560	3.8	
	North America	577	0.3	571	0.3	
	South America	7,049	3.5	6,958	3.5	
	Swedish-migrant	37,917	18.8	37,605	18.8	
	Mixed migrant	12,952	6.4	12,846	6.4	
	Swedish-born, no parents	254	0.1	0	0.0	

[a] Region of birth for migrants, region of parental birth for children of migrants; [b] Includes Children of migrants with one Nordic-born parent and one Swedish-born parent; [c] Chi-squared test, degrees of freedom, p-value

Table 2 Distribution of the migrant density proportion across neighbourhoods

	Mean	SD	Median	IQR		Min	Max
				25%	75%		
Own-region migrant density	0·104	0·115	0·064	0·030	0·130	0·0001	0·800
Generation-specific density (migrants)	0·079	0·084	0·049	0·022	0·106	0·0001	0·587
Generation-specific density (children of migrants)	0·044	0·043	0·034	0·015	0·056	0·0001	0·355
Visible minority density	0·251	0·214	0·183	0·070	0·387	0·0012	0·947

Table 3 Percent (%) migrants & children of migrants in each quintile of own-group migrant density, by region

	Quintile 1	Quintile 2	Quintile 3	Quintile 4	Quintile 5
Own-group (overall)	17·6	20·3	20·6	20·7	20·7
Nordic	0·3	12·2	36·5	36·6	14·3
Europe	5·2	18·0	24·6	28·5	23·8
Asia	32·2	28·6	16·2	17·0	6·0
Oceania	100·0	0·0	0·0	0·0	0·0
Middle East + North Africa	11·9	13·9	12·2	18·8	43·2
sub-Saharan Africa	32·7	21·1	14·5	14·3	17·4
North America	92·2	4·3	2·2	1·4	0·0
South America	48·6	32·4	14·5	3·9	0·6
Swedish-migrant	16·6	55·4	27·3	0·8	0·0
Mixed migrant	95·1	4·9	0·0	0·0	0·0

Table 4 Neighbourhood level random effects

Neighbourhood-level random effects	Sigma	95% CI		p-value
Null model	0·04	0·02	0·08	0·0001
Unadjusted model	0·05	0·03	0·08	<0·001
Individual-adjusted model	0·07	0·04	0·11	<0·001
Neighbourhood-adjusted model	0·03	0·02	0·07	<0·001
Fully adjusted	0·06	0·04	0·10	0·001

Table 5 Migrant density by generation and region

	Migrants				Children of migrants			
	aHR[a]	95%	CI	AIC	aHR[a]	95%	CI	AIC
Adjusted + region								
Quintile 1 (lowest)	1·30	1·08	1·56[*]	36,011	1·03	0·83	1·27	33,240
Quintile 2	1·12	0·95	1·32		1·01	0·85	1·20	
Quintile 3	1·05	0·89	1·22		0·98	0·84	1·15	
Quintile 4	1·08	0·93	1·24		0·94	0·81	1·09	
Quintile 5 (highest - ref·)	1				1			
5% decrease	1·03	1·00	1·06[d]	36,011	1·00	0·97	1·03	33,235
Neighbourhood random effect	0·13	0·09	0·20		0·44	0·40	0·48	

HR: Hazard ratio; 95%CI: 95% confidence interval
[*] p<0·05
[a] Individual + family confounders: age, sex, lone dwelling, family income, social welfare, unemployment, lone dwelling, and time since migration (migrants only)· Neighbourhood confounders: deprivation index, population density, and proportion lone dwelling
[b] Includes children of migrants with one Nordic-born parent and one Swedish-born parent
[c] Numbers were too low in Oceania group for the model to converge, so excluded from this analysis
[d] p=0·04

Table 6 Strengthening the Reporting of Observational Studies in Epidemiology (STROBE) checklist for cohort studies

	Item No	Recommendation	
**Title and abstract**	1	(*a*) Indicate the study’s design with a commonly used term in the title or the abstract (pages 1 & 5)	☑
		(*b*) Provide in the abstract an informative and balanced summary of what was done and what was found (page 5)	☑
**Introduction**			
Background/rationale	2	Explain the scientific background and rationale for the investigation being reported (pages 7-8)	☑
Objectives	3	State specific objectives, including any prespecified hypotheses (page 8)	☑
**Methods**			
Study design	4	Present key elements of study design early in the paper (pages 8-9)	☑
Setting	5	Describe the setting, locations, and relevant dates, including periods of recruitment, exposure, follow-up, and data collection (pages 8-9)	☑
Participants	6	(*a*) Give the eligibility criteria, and the sources and methods of selection of participants. Describe methods of follow-up (pages 8-9)	☑
Variables	7	Clearly define all outcomes, exposures, predictors, potential confounders, and effect modifiers. Give diagnostic criteria, if applicable (pages 9-10)	☑
Data sources/ measurement	8*	For each variable of interest, give sources of data and details of methods of assessment (measurement). Describe comparability of assessment methods if there is more than one group (pages 9-10)	☑
Bias	9	Describe any efforts to address potential sources of bias (page 11)	☑
Study size	10	Explain how the study size was arrived at (Figure 1, supplement Table 1)	☑
Quantitative variables	11	Explain how quantitative variables were handled in the analyses. If applicable, describe which groupings were chosen and why (pages 9-10)	☑

Statistical methods	12	(*a*) Describe all statistical methods, including those used to control for confounding (pages 10-11)	☑
		(*b*) Describe any methods used to examine subgroups and interactions (page 11)	☑
		(*c*) Explain how missing data were addressed (page 12)	☑
		(*d*) If applicable, explain how loss to follow-up was addressed	NA
		(*e*) Describe any sensitivity analyses	NA
**Results**			
Participants	13*	(a) Report numbers of individuals at each stage of study—eg numbers potentially eligible, examined for eligibility, confirmed eligible, included in the study, completing follow-up, and analysed (Figure 1)	☑
		(b) Give reasons for non-participation at each stage (Figure 1)	☑
		(c) Consider use of a flow diagram (Figure 1)	☑
Descriptive data	14*	(a) Give characteristics of study participants (eg demographic, clinical, social) and information on exposures and potential confounders (Table 1)	☑
		(b) Indicate number of participants with missing data for each variable of interest (Table 1)	☑
		(c) Summarise follow-up time (eg, average and total amount) (page 11, Table 1 & 4)	☑
Outcome data	15*	Report numbers of outcome events or summary measures over time (Table 1)	☑
Main results	16	(*a*) Give unadjusted estimates and, if applicable, confounder-adjusted estimates and their precision (eg, 95% confidence interval). Make clear which confounders were adjusted for and why they were included (Table 2)	☑
		(*b*) Report category boundaries when continuous variables were categorized (Table 2)	☑
		(*c*) If relevant, consider translating estimates of relative risk into absolute risk for a meaningful time period	NA
Other analyses	17	Report other analyses done—eg analyses of subgroups and interactions, and sensitivity analyses (Table 3-4, Supplemental tables)	☑
**Discussion**			
Key results	18	Summarise key results with reference to study objectives (page 14)	☑
Limitations	19	Discuss limitations of the study, taking into account sources of potential bias or imprecision. Discuss both direction and magnitude of any potential bias (pages 14-15)	☑
Interpretation	20	Give a cautious overall interpretation of results considering objectives, limitations, multiplicity of analyses, results from similar studies, and other relevant evidence (pages 16-18)	☑
Generalisability	21	Discuss the generalisability (external validity) of the study results (pages 16-18)	☑
**Other information**			
Funding	22	Give the source of funding and the role of the funders for the present study and, if applicable, for the original study on which the present article is based (page 6)	☑

*Give information separately for exposed and unexposed groups.
